# Assessment of Compressive Mechanical Behavior of Bis-GMA Polymer Using Hyperelastic Models

**DOI:** 10.3390/polym11101571

**Published:** 2019-09-27

**Authors:** Atefeh Karimzadeh, Majid Reza Ayatollahi, Seyed Saeid Rahimian Koloor, Abd Razak Bushroa, Mohd Yazid Yahya, Mohd Nasir Tamin

**Affiliations:** 1 Centre of Advanced Composite Materials, Universiti Teknologi Malaysia, Johor Bahru 81310, Malaysia; a.karimzadeh.66@gmail.com (A.K.);; 2 Fatigue and Fracture Laboratory, Center of Excellence in Experimental Solid Mechanics and Dynamics, School of Mechanical Engineering, Iran University of Science and Technology, Tehran 16846, Iran; 3 Department of Aerospace Engineering, Faculty of Engineering, Universiti Putra Malaysia, UPM Serdang 43400, Selangor Darul Ehsan, Malaysia; 4 Institute for Nanomaterials, Advanced Technologies and Innovation, Technical University of Liberec, Studentska 2, 461 17 Liberec, Czech Republic; 5 Department of Mechanical Engineering, Faculty of Engineering, University of Malaya, Kuala Lumpur 50603, Malaysia; 6 Centre of Advance Manufacturing and Mechanical Engineering, Faculty of Engineering, University of Malaya, Kuala Lumpur 50603, Malaysia; 7 School of Mechanical Engineering, Universiti Teknologi Malaysia, Johor Bahru 81310, Malaysia

**Keywords:** Bis-GMA polymer, hyperelastic constitutive model, compressive behavior, finite element method, nano-indentation experiment

## Abstract

Despite wide industrial applications of Bis-GMA polymer, very few studies are available about the material classification, mechanical properties, and behavior of this material. In this study, the compressive behavior of Bis-GMA polymer was studied using different hyperelastic constitutive models through a hybrid experimental-computational process. Standard uniaxial compression tests were conducted to extract the mechanical behavior and structural response of the Bis-GMA polymer. A nano-indentation experiment was used to verify the compressive behavior of Bis-GMA polymer in the form of hyperelastic behavior. The finite element model and real-time simulation of the test incorporating different hyperelastic models were developed in comparison with the experimental finding to obtain the proper type of hyperelastic behavior of Bis-GMA polymer. The results indicate that a second-order polynomial hyperelastic model is the best fit to predict the behavior of Bis-GMA polymer. Next, the validated model was used to determine the true stress–strain curve of the Bis-GMA polymer.

## 1. Introduction

Polymer materials are rapidly used in the manufacturing of advanced structures for various industrial applications from biomechanics to aerospace, etc. [1]. The characterization of the properties and mechanical behavior of polymeric materials have been considered as great challenges in the design and analysis of novel composites and polymer-based structures [2,3,4,5,6]. Various types of polymer materials have been synthesized with brittle [3], ductile [4], hyperelastic [6], etc., behaviors for different mechanical applications. Among the various models recommended for the mechanical behavior of polymers, the hyperelastic model is generally employed for modelling the nonlinear elastic behavior of homogeneous and isotropic polymers that exhibit large deformation [7,8,9,10]. The hyperelastic behavior of polymers is normally examined through their reversible behavior in the loading-unloading process, in which a nano-indentation experiment is also used to assess the nonlinear elastic behavior of the polymers through the loading-unloading [11,12,13,14]. 

Many hyperelastic models have been derived to describe the mechanical behavior of polymeric materials, including Ogden, Arruda and Boyce, Polynomial, Van Der Waals, and Yeoh models [6,15,16,17,18]. Khalajmasoumi et al. [6] determined the behavior of polyethylene under a monotonic compressive load using the Yeoh hyperelastic model. This model is derived from the Polynomial model and developed for characterizing the behavior of hard polymers [6]. The Arruda and Boyce model was applied by Ayoub et al. [18] and then Zhang and To [16], to predict the hyperelastic behavior of hard polymeric materials. However, the assessment of the appropriate hyperelastic models for the accurate prediction of the linear-nonlinear mechanical behavior of polymers is a challenging matter dependent on the chemical composition and physical response of the polymer as well as on the fundamental understanding of the mathematical formulation, etc. [9,17,18,19]. In some cases, a hybrid experimental-computational approach was developed to quantify the mechanical behavior of the polymers with respect to the application load and boundary conditions [20,21]. The hybrid approach normally incorporates a mathematical-physical model with different variables into a three-dimensional (3D) model of the test sample in an iterative procedure to examine and validate the constitutive model [22,23]. The computational process is normally implemented using the finite element (FE) method, which is recognized as one the useful methods to simulate the linear-nonlinear elastic behavior of polymers [6,14,16,17,24]. 

Bisphenol A diglycidyl dimethacrylate or Bis-GMA polymer has been used in many applications, including green composites, bone cements, dental restorative composites and cements, dentine bonding agents, pit and fissure sealants, elastomers, dental adhesives, etc. [25,26,27,28,29,30,31,32,33,34]. In green composites, Bis-GMA is considered as the matrix constituent of the composite that was reinforced using amine functionalized paper pulp [25]. The polymerization characteristics of Bis-GMA cause the formation of a three dimensional crosslinking, which results in a tight structural binding with the filler materials [27]. These characteristics of Bis-GMA polymer, along with the bioactivity feature, makes this polymer appropriate to serve as an organic matrix of dental composites and bone cements [27,35]. The physical and mechanical properties of different combinations of Bis-GMA and other monomers such as TEGDMA has been investigated in previous research [36,37,38,39,40]. The mechanical properties of Bis-GMA polymer, including the hardness, diametral tensile strength, flexural modulus and strength, compressive strength, and Young’s modulus have been reported in previous studies [25,41]. 

In many applications, Bis-GMA polymer in the form of a matrix constituent of composites or standalone, were used to bond mesoscale parts as an adhesive, sealant to block defects, restoration, laminating and veneering of bodies, etc. In these applications, Bis-GMA polymer is normally subjected to continuous load under compressive deformation [26,42,43,44,45]. Despite the wide applications of Bis-GMA polymer, very little study is available on the examination of the material classification, as well as the mechanical properties and behavior under monotonic compressive load. Furthermore, no specific constitutive model is prescribed for the mechanical behavior of Bis-GMA polymer. Therefore, this study aims to assess the compressive mechanical behavior of Bis-GMA polymer using various hyperelastic constitutive models. In this respect, several nano-indentation tests are performed to examine the hyperelasticity and reversible behavior of the Bis-GMA polymer through the loading-unloading behavior. A set of standard compression tests is conducted on Bis-GMA polymer to measure the mechanical response and structural deformation of the test samples. A hybrid experimental-computational approach is developed to determine the suitable hyperelastic constitutive model for Bis-GMA polymer through the comparison of the mechanical behavior as well as the structural deformation. The proper constitutive model, the elastic limit of the stress–strain curve, as well as the mechanical property and behavior of Bis-GMA polymer, are determined. In addition, the results are used to characterize the true stress–strain behavior of the Bis-GMA polymer. 

## 2. Hyperelastic Constitutive Model

The mechanical behavior of hyperelastic materials is normally defined using strain energy potential, that is the energy stored in the material per unit volume as a function of strain in local material point [46]. This energy potential is derived based on deviatoric and volumetric components as:

(1) $$U=U_{dev}({\bar{I}}_{1},{\bar{I}}_{2})+U_{vol}(J_{el})$$

Many forms of strain energy potential are derived for polymers including Arruda–Boyce [47], Polynomial [48], Reduced polynomial [49], Ogden [50], Yeoh [51], Van der Waals [52], etc. These models are used to simulate the Bis-GMA polymer behavior, in which the energy potential formulation is listed in Table 1. In these equations, *U* is the strain energy density, *C*, *D*, *μ*, and *λ_m_* are the material parameters, *J_el_* is the elastic volume ratio, ${\bar{I}}_{1}$ and ${\bar{I}}_{2}$ are the first and second deviatoric strain invariants, which with the assumption of full incompressibility, are defined as [6,53,54]:

(2) $$J_{el}={\left(\lambda_{1}\lambda_{2}\lambda_{3}\right)}_{el}=1$$

(3) $${\bar{I}}_{1}=\lambda_{1}^{2}+\lambda_{2}^{2}+\lambda_{3}^{2}$$

(4) $${\bar{I}}_{2}=\lambda_{1}^{\left(-2\right)}+\lambda_{2}^{\left(-2\right)}+\lambda_{3}^{\left(-2\right)}$$

where *λ*_1_, *λ*_2_ and *λ*_3_ are the principal stretches and the subscript *el* refers to the elastic limit. The detailed information about the equations and models could be found elsewhere [47,48,49,50,51,52]. 

The stress–strain behavior of Bis-GMA polymer that was obtained through experiment is used to specify an appropriate hyperelastic model. The constitutive model for representing the hyperelastic behavior of the Bis-GMA polymer was initially unknown. Several potential hyperelastic models were selected (Table 1), in which the relevant models capable of predicting the local and global hyperelastic behaviors of the Bis-GMA polymer were specified to reduce the trivial iterations. Hence, in the first step of the simulation, the response of each hyperelastic model was evaluated by simulating the local behavior of the Bis-GMA polymer under uniaxial compression load. The structural response of the Bis-GMA polymer was then investigated through the FE simulation of the compression test to determine the type of hyperelastic constitutive model. In this respect, the material response of the Bis-GMA polymer obtained from each hyperelastic model was compared with the uniaxial compression test data through a curve fitting process. A least-squares fitting method was used to minimize the error function *E* for the ‘*n*’ number of stress and strain pair points that were obtained from the first step of the FE simulation and the compression test data. The function is given as:

(5) $$E=\sum_{i=1}^{n}{\left(1-\frac{T_{i}^{th}}{T_{i}^{test}}\right)}^{2}$$

where $T_{i}^{th}$ is the theoretical stress expression obtained from the constitutive models and $T_{i}^{test}$ is a stress value obtained from the experimental data. In the uniaxial tests, the theoretical stress ($T^{th}$) can be calculated from the uniaxial stress (*T_u_*), which is derived from the strain energy density by applying the principle of virtual work as follows:

(6) $$T_{u}=\frac{\delta U}{\delta \lambda_{u}}$$

where *λ_u_* is the stretch in the loading direction. 

## 3. Material and Experiment Method

A set of uniaxial compression tests was performed on Bis-GMA polymer to investigate the compressive mechanical behavior using the hyperelastic model. The nominal stress–strain curve and the approximate maximum compressive strain in which the material behaves elastically were determined through the experiment. In addition, the hyperelastic behavior of the Bis-GMA polymer within the strain range that assumed as the hyperelastic limit in the compression test, was examined through loading-unloading curves acquired from the nano-indentation experiment. In the nano-indentation process, the material beneath the indenter tip is under compressive load, therefore this experiment could be valid to investigate the compressive behavior of the polymer [55].

### 3.1. Sample Preparation

Bis-GMA polymer is made photo-polymerizable, to form the shape of specimens for compression test through curing under a light source. For this purpose, Bis-GMA is often combined with camphoroquinone (CQ) and dimethylamine ethyl methacrylate (DMAEMA) [36,56]. The chemical characteristics of the materials used for preparing the polymer are presented in Table 2. To make a photo-polymerizable Bis-GMA, it was heated to 50 °C to reduce the viscosity for better mixing with the photo-initiators. Then, a 0.4 mol % CQ and 0.8 mol % DMAEMA were added to Bis-GMA and blended [36,56]. The mixture was stored in a dark container before casting. Two sets of specimen were prepared for the compression experiment and nano-indenteation test.

The specimens of the compression test were prepared using a few numbers of translucent molds with a 5 mm-diameter and 11 mm-height. The internal walls of the molds were lubricated carefully with oil for more convenient detachment of the polymer after polymerization. Next, the photo-polymerizable Bis-GMA was injected into the mold at a temperature of 50 °C and cured with a 400 W/m^2^ LED light for 60 s from each side (i.e., top, bottom, and surrounding). In the next step, all of the cured Bis-GMA specimens were ejected from the mold. The top and bottom surfaces of the specimens were smoothened with 400–2000 grit abrasive papers. The final height of all samples was 10 ± 0.05 mm. 

The nano-indentation experiment were performed on a disk shape specimen with a diameter of 5 mm and height of 4 mm, prepared using the same procedure as the specimen of the compression test. Since the nano-indentation experiment requires a very smooth surface, the surface of the specimen was ground with 400–2500 grit abrasive papers and polished using diamond paste with 1 and 0.5 micron mesh sizes.

### 3.2. Compression Test Method

The uniaxial compression experiment was performed on the Bis-GMA specimens with a displacement rate of 1 mm/min according to ASTM D695 standard [57] at room temperature using a universal testing machine (Instron, Norwood, MA, USA). The top and bottom surfaces of all samples were lubricated to minimize the friction coefficient between the sample surfaces and the compression discs. The experiment was repeated on 8 specimens, and the load-deformation response of the each specimen was recorded throughout the tests. During the loading, each specimen was monitored carefully to detect an approximate limit for the hyperelastic behavior. It was observed that the polymer discoloration occurred in the specimen at the center and near its top and bottom surfaces. Then, some small voids like crazes were generated at the discolored area where damage could be observed visually. The machine crosshead displacement level corresponding to the observed onset of discoloration was recorded as the limit of the hyperelastic behavior. The corresponding local material displacement was determined later through the hybrid-experimental-computational approach.

### 3.3. Nano-Indentation Test Method

The nano-indentation experiment has been recommended as one of the methods to examine the elastic behavior of polymeric materials [11,12,14]. In this respect, nano-indentation experiments were carried out to examine the hyperelasticity of the Bis-GMA polymer through loading-unloading processes using a Triboscope test system (Hysitron Inc., Minneapolis, MN, USA) and a Berkovich indenter. The tests were implemented based on ISO 14577 standard [58], and the loading was set such that the polymer loaded within the hyperelastic strain range. The experiments were performed in the load-control mode with two steps of loading and unloading processes at the indenter displacement rates of 1 mm/min for the duration of 60 s. The indentation load level was set to a value such that the maximum indentation strain was at the hyperelastic strain limit of 0 to 0.3. The indentation strain was calculated through a validated finite element simulation (FES) of the nano-indentation experiment, which was performed according to the method described in a previous study [59]. However, in this study the material was considered to be hyperelastic, in which the nominal stress–strain curve was obtained from the compression test and used in the finite element model. 

## 4. Finite Element Simulation

**Figure 1:**
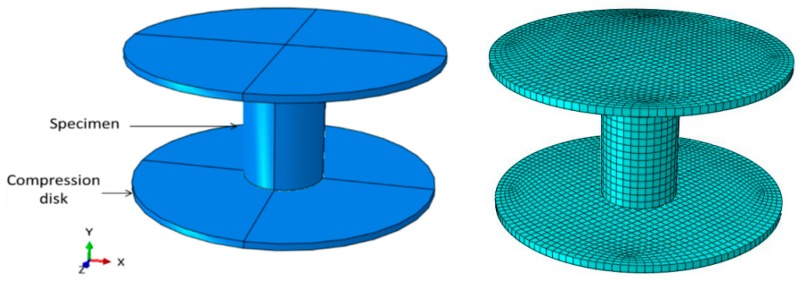
Geometry (**left**) and mesh configuration (**right**) of FE model representing the Bis-GMA polymer under compression loading condition.

The compressive behavior of Bis-GMA polymer under uniaxial compression load was simulated in Abaqus 6.14 commercial software (Dassault Systèmes, Vélizy-Villacoublay, France) [49] using hyperelastic models that was applied on a three-dimensional finite element model of the test samples. A deformable body was created for the Bis-GMA polymer specimen and two discrete rigid circular disks as the compression disks were considered, as shown in Figure 1 (left). The Bis-GMA polymer was modeled using 8-node linear brick, reduced integration, hourglass control (C3D8R) element, while the rigid body discs were meshed using 4-node 3-D bilinear rigid quadrilateral (R3D4) element. The mesh configuration of the specimen and the loading disks are shown in Figure 1 (right). The non-linear geometry option was also assigned to the deformable cylindrical specimen. 

The nominal stress–strain curve within the limit of hyperelastic behavior obtained from compression test, as described in Section 3.2, was used in the computational process. The Poisson’s ratio value of the Bis-GMA polymer was assumed 0.4 as reported in previous studies [60,61,62] that was used as input data in the FE model. 

The contact condition between the rigid body disks and the sample was defined as a frictionless surface to surface contact with the finite sliding condition. The boundary conditions were specified similar to the test conditions, i.e., the lower compression disk was fixed in all directions and the upper disk was allowed to move downward along the sample axial direction. The displacement of the upper compression disk was set to the maximum displacement of 3 mm representing the elastic behavior of the Bis-GMA polymer as detected for the hyperelastic limit during the experiment. The bottom central point of the cylinder cross section was fixed in order to prevent any sliding in the x and z directions. 

A mesh convergence study was performed to minimize the effect of the element size on the computation of the field variables and the global response of the polymer. The element size of the specimen was refined until the variation of the predicted axial stress and load-displacement was saturated. A total number of 2200 elements were found to be sufficient for the simulation process. 

## 5. Hybrid Experimental-Computational Approach

**Figure 2:**
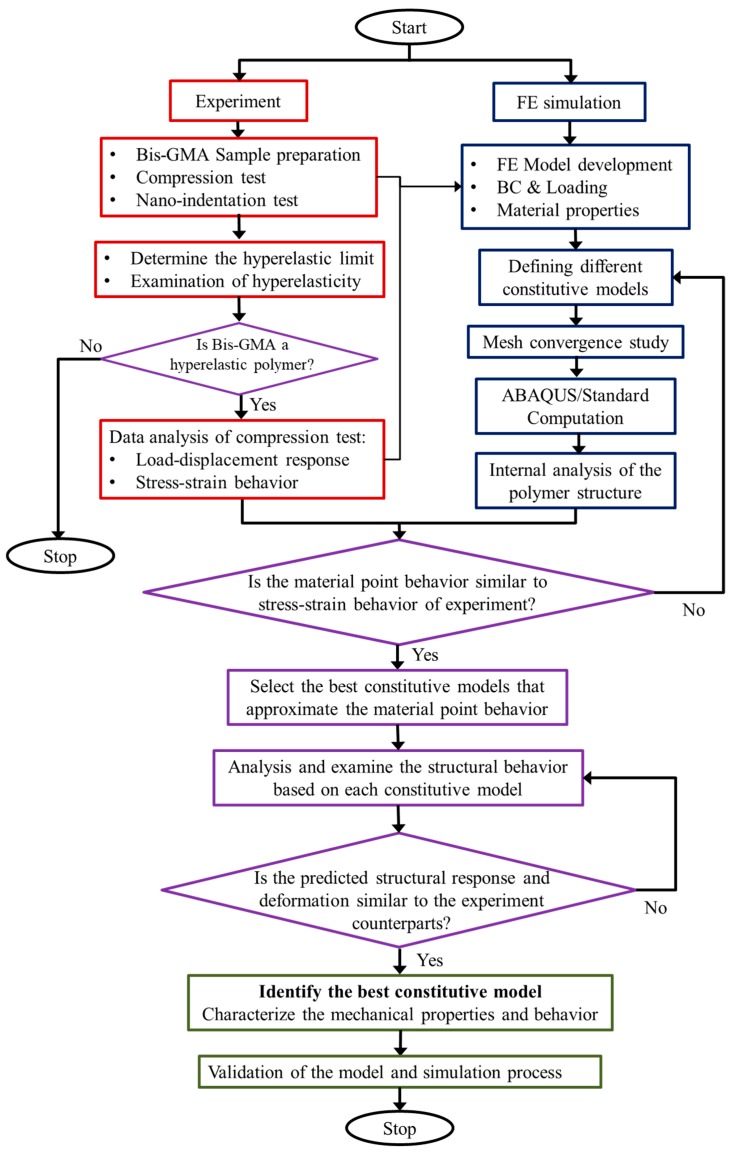
Flowchart of the hybrid experimental-computational approach to determining the mechanical behavior of hyperelastic polymers.

A hybrid experimental-computational approach was developed to examine the predictability of the compressive behavior of Bis-GMA polymer using hyperelastic models, in which the flowchart of the process is shown in Figure 2. The approach logically links the limited experiment data to the simulation results and determines the constitutive model and mechanical properties of Bis-GMA polymer. In the first step (experiment), a set of uniaxial compression tests on standard Bis-GMA polymer sample was implemented, in which the load-deformation response was used to determine the nominal stress–strain behavior. The limit of the hyperelastic response of the polymer was determined through the compression test. An additional set of nano-indentation experiments was performed on the Bis-GMA polymer to double-check the elastic behavior through loading-unloading process. The study was supposed to stop if the hyperelastic behavior was not determined for the Bis-GMA polymer. In the second step (FE simulation), a 3D FE model of the polymer sample was developed, which is used to assess the mechanical behavior and mechanism of deformation of Bis-GMA polymer based on different hyperelastic constitutive models.

The best constitutive model is specified after two steps of examination.^.^ First, by selecting a group of constitutive models that approximate the material point behavior similar to the nominal stress–strain obtained from the experiment, and second, through the internal analysis of the FE model based on the selected models (first round), to examine the accuracy of the predicted results in terms of structural response and deformation of the polymer sample. Once the best hyperelastic constitutive model is identified, the mechanical characterization of the Bis-GMA polymer is completed. In the final step, the hybrid approach leads to attaining a validated FE model and simulation process. The hybrid approach is recommended to be used for the mechanical characterization and the prediction of the mechanical behavior of other hyperelastic polymers under different quasi-static monotonic loads.

## 6. Results and Discussion

The results are presented in three subsections, first to illustrate the test results of the Bis-GMA polymer under compressive load in terms of load-deformation and compressive stress–strain curves. Then, the elastic behavior of the Bis-GMA polymer in nonlinear form are investigated using the nano-indentation experiment. In the last subsection, the FE simulation results of Bis-GMA polymer using various hyperelastic models are illustrated in comparison with experiment data. A comprehensive discussion is given for the assessment and selection of a validated hyperelastic model to predict the compressive behavior of Bis-GMA polymer.

### 6.1. Compression Test Results

**Figure 3:**
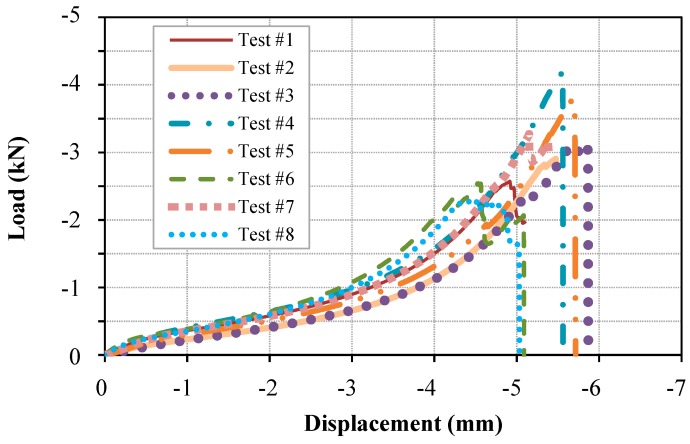
Load-displacement response of uniaxial compression test on Bis-GMA polymer.

The load versus load-line displacement curves obtained from the compression tests on the Bis-GMA polymer samples are illustrated in Figure 3. As recommended by ASTM D695 standard, more than five samples were tested to obtain an acceptable average response of the structure. A very close load-displacement response was measured for the samples up to 3 mm displacement (Tests No. 1, 4, 7, 8), with 0.14 kN standard deviation in load value with respect to the average value of 0.87 kN. The limit of hyperelastic behavior (refer to Section 3.2) was determined at the average displacement of 3 mm with the standard deviation of 0.25 mm. 

**Figure 4:**
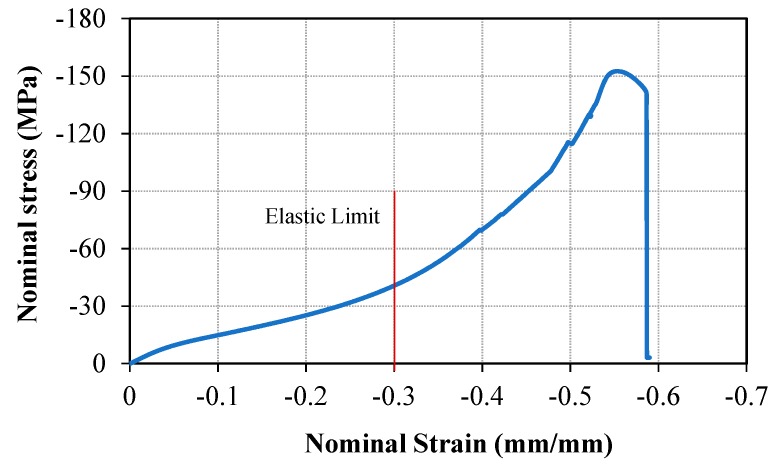
The mean values of nominal stress versus nominal strain obtained from the experimental results.

The nominal stress–strain behavior of Bis-GMA polymer was obtained for each test, in which the stress and strain values were calculated directly from the force and deformation values measured in each test and from the geometry of the sample in the respected test. Next, the stress–strain behavior of Bis-GMA polymer was obtained from the average behavior of the samples, as illustrated in Figure 4. The average elastic limit was obtained up to 0.3 compressive strain. This hyperelastic curve was used to define the material property of Bis-GMA polymer in the FE simulation.

### 6.2. Examination of Bis-GMA Polymer Hyperelastic Behavior Through Nano-Indentation Test 

**Figure 5:**
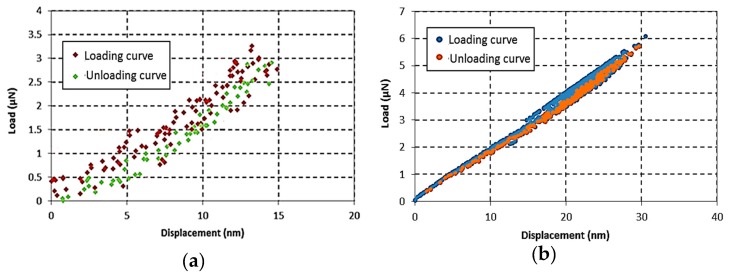
The loading-unloading responses of Bis-GMA polymer in the nano-indentation test (indentation loads of 3 μN (**a**) and 6 μN (**b**)).

The hyperelstic limit of Bis-GMA polymer was obtained up to a 0.3 compressive strain (Figure 4). The similar strain range was prescribed for the nano-indentation test on the Bis-GMA polymer. The results of the nano-indentation tests were recorded as a force-displacement curve during the loading-unloading process as shown in Figure 5. Results were shown for two different indentation loads of 3 μN and 6 μN corresponding to the indentation strains of 0.2 and 0.3 [63], respectively. The relatively higher scattered data observed in the load-displacement curve of test No. 1 is due to the very low indentation load applied to a rather soft specimen. This problem was resolved in test No. 2 by applying a higher indentation load. The similar trace of loading and unloading curves indicates the elastic deformation of the material within the prescribed load level. Moreover, the gradual diminishing value of the reaction force through the unloading process confirms the absence of any plastic strain in the Bis-GMA polymer [11,64]. It can be concluded that the results of the nano-indentation experiments and also the nonlinear form of the load-displacement curves obtained from the compression tests (Figure 3 and Figure 4), confirm that the Bis-GMA polymer behaves as hyperelastic materials under compressive load.

### 6.3. FE Simulation Results

#### 6.3.1. Initial Selection of the Proper Hyperelastic Models

**Figure 6:**
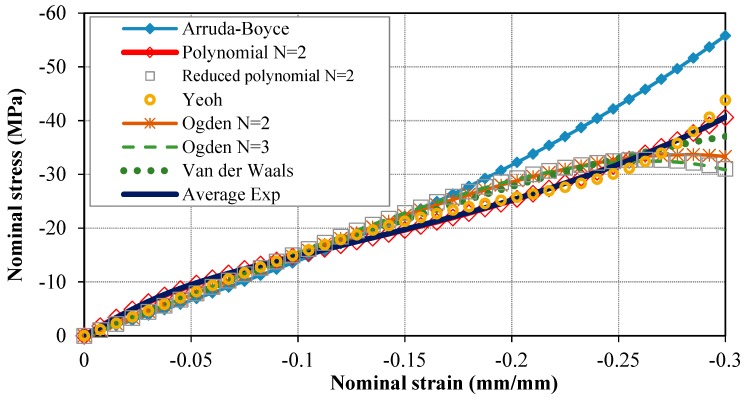
The resultant stress–strain curves that fitted using the hyperelastic models provided in Table 2.

The results of local material behavior in terms of the stress–strain curve obtained from different hyperelastic models (refer to Section 2) are presented in Figure 6. The Bis-GMA polymer behavior was predicted closely by the FE model that used second order polynomial (i.e., polynomial N = 2), Van der Waals, and Yeoh hyperelastic models. Therefore, these three hyperelastic models were considered as the appropriate models for further investigation. 

#### 6.3.2. Prediction of the Bis-GMA Polymer Structural Response 

**Figure 7:**
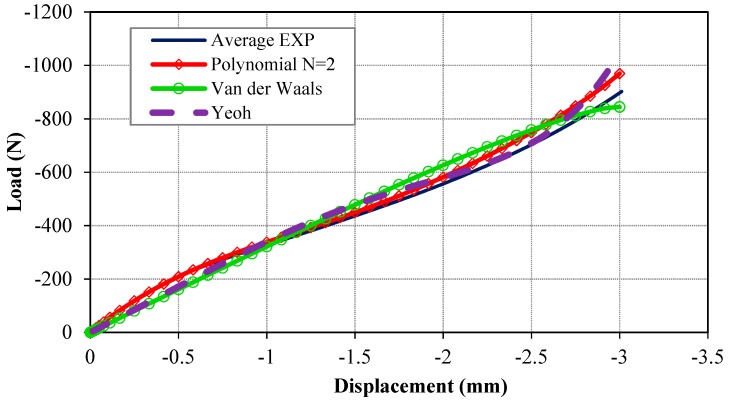
The predicted load-displacement curves and stress–strain responses of the system in comparison with the experimental data.

The results of the FE model incorporating the selected hyperelastic models of second order polynomial (i.e., polynomial N = 2), Van der Waals, and Yeoh are presented to describe the capability of each model in prediction of the deformation and the response of the Bis-GMA polymer under compressive load. The average load-displacement responses of the Bis-GMA polymer samples were plotted along with its computed counterparts, as shown in Figure 7. A very similar trend of the experiment data is predicted by the polynomial N = 2 model, while inconsistent results are predicted by the other models. The maximum difference between the results of the polynomial model is less than those of the Van der Waals and Yeoh models. The polynomial model predicted an acceptable response of the experiment with maximum error of 2, 3.5, and 7% at 1, 2, and 3 mm displacement, respectively. The average error of the response by polynomial model in comparison with the measured data of many tests (Figure 3) is negligible. 

#### 6.3.3. Prediction of the Bis-GMA Polymer Structural Deformation

**Figure 8:**
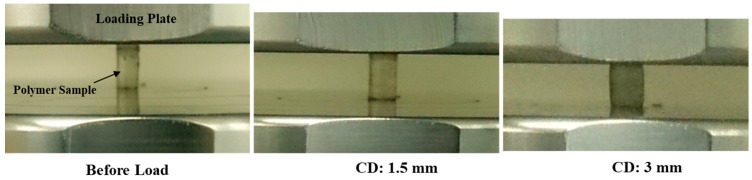
Structural deformation of the Bis-GMA polymer specimen under different compressive deformation (CD).

The structural deformation of Bis-GMA polymer sample at different levels of compressive displacement is shown in Figure 8. As the polymer cylinder was compressed, 0.82 mm average expansion was measured in the transverse direction at the center of the outer surface of the polymer cylinder while it was loaded up to 3 mm deformation. The diameter of the central section of the specimen was expanded slightly more than the top and bottom surfaces.

**Figure 9:**
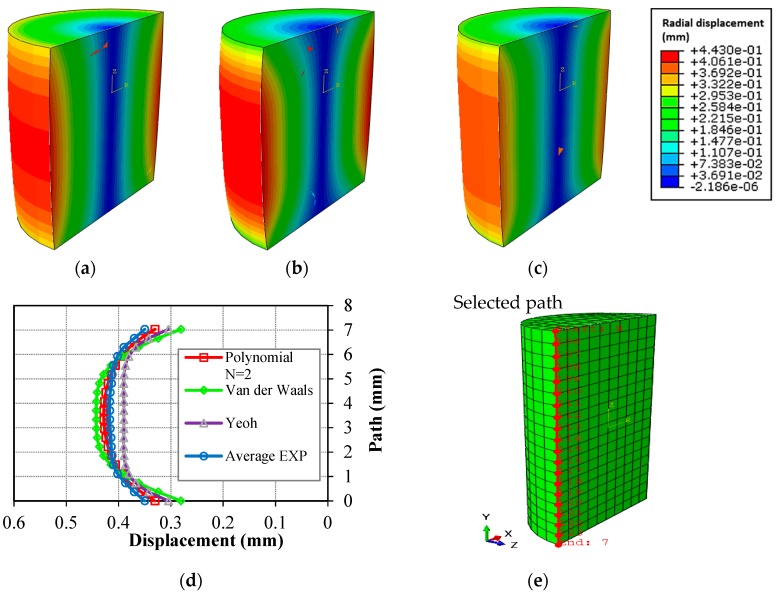
Contour plots of the radial deformation of the FE model incorporating polynomial N = 2 (**a**), Van der Waals (**b**) and Yeoh (**c**) hyperelastic models, and plot of transverse deformation (**d**) of a selected path (**e**) on the FE models.

Previous studies have indicated that a proper prediction of structural deformation of the test samples under compressive load could be considered as one of the important factors in the selection process of the constitutive model [6,65]. In this respect, the structural deformation of the FE model of the polymer sample with polynomial N = 2, Van der Waals, and Yeoh hyperelastic models is shown in Figure 9a–c. The results are presented in term of contour plot of radial deformation at 3 mm compressive displacement. It is shown that, considering different hyperelastic models, different shapes are predicted for the structural deformation. In the case where Yeoh model was applied, the maximum diameter expansion of 0.74 mm was obtained which is less than the corresponding value measured in the experiments. The maximum diameter expansions of 0.88 and 0.86 mm were obtained using the Van der Waals and polynomial N = 2 models, respectively, which are close to the diameter expansion range of the sample in the experiment (0.82 mm). The physical deformation of the sample during the test (Figure 9) was predicted more likely using the polynomial N = 2 and Van der Waals models. A better view of such interpretation could be presented by plotting the transverse expansion of the specimen (at 3 mm compressive displacement), as shown in Figure 9d,e. Results indicated a very close structural deformation with a similar trend by the polynomial model with respect to the experiment data. The Yeoh and Van der Waals models prediction of the central transverse expansion and the trend of the deformation curve mismatched the structural deformation. Therefore, the polynomial N = 2 model could be considered as the valid model to predict the structural deformation of the Bis-GMA sample. 

#### 6.3.4. Identification of the Best Hyperelastic Model

The second order polynomial model (i.e., polynomial N = 2) was identified as the best hyperelastic model that predict the local material behavior, structural response as well as the deformation of the Bis-GMA polymer structure, characterized through the hybrid experimental-computational approach. The constitutive equation of the polynomial N = 2 model can be obtained by expanding the general polynomial form equation [48] in Table 2 for *N* = 2 which gives:

(7) $$\begin{array}{l} U=C_{01}({\bar{I}}_{2}-3)+C_{10}({\bar{I}}_{1}-3)+C_{11}({\bar{I}}_{1}-3)({\bar{I}}_{2}-3)+C_{02}{\left({\bar{I}}_{2}-3\right)}^{2}+C_{20}{\left({\bar{I}}_{1}-3\right)}^{2} \end{array}$$

where *U* is the strain energy per unit of reference volume. The parameters ${\bar{I}}_{1}$ and ${\bar{I}}_{2}$ (presented in Equations (2) to (4)) were calculated for the uniaxial compression behavior up to the displacement of 3 mm. In Equation (7), *C_ij_* s are hyperelastic material constants which were calculated by a curve fitting process, as described in Section 2. These material constants were determined in this study for the Bis-GMA polymer as:

$$C_{01}=-259.62MPa,\;C_{10}=300.35MPa,\;C_{11}=279.21MPa,\;C_{02}=-187.68MPa,\;C_{20}=81.33MPa.$$

As mentioned earlier, Bis-GMA polymer is frequently used as a matrix of dental restorative composites, constituent of bone cements, adhesives, etc., restoration, etc. [26,42,43,44,45]. However, to interpret the mechanical behavior of these materials, a better understanding of the Bis-GMA polymer behavior is necessary [28,66,67]. Since Bis-GMA shows a hyperelastic behavior under compression loading, the assumption of a linear stress–strain relation cannot accurately describe its real mechanical behavior. 

### 6.4. Determination of the Bis-GMA Polymer True Stress–Strain Curve 

**Figure 10:**
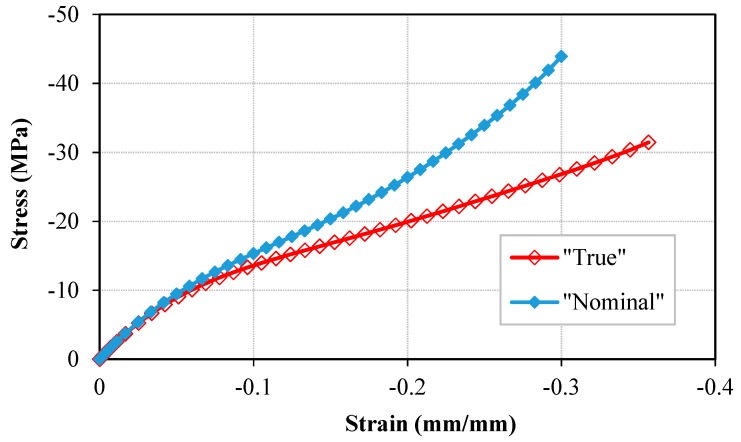
The true stress–strain curves obtained from the structural response of the model using different hyperelastic equations.

Determination of the true stress–strain behavior of polymers is important for the investigation on the mechanical behavior of polymer materials and structures [68,69]. It is difficult to obtain the true compressive stress during an experiment due to the difficulties in measurement of the variation in the instantaneous cross-sectional area throughout the loading. In this study, the hybrid method was used to identify the best constitutive model to predict the material response as well as the structural deformation. Therefore, the result of the validate FE model is used to measure the variation of the specimen cross-sectional area throughout the loading. Subsequently, the true stress–strain curve of the Bis-GMA polymer is obtained as plotted in Figure 10. The results indicate a similar curve to the nominal stress–strain behavior, with slightly lower values as the cross-sectional area of the sample continuously increases while the specimen is compressed.

## 7. Conclusion 

The compressive mechanical behavior of Bis-GMA polymer was examined with the assumption of hyperelastic behavior. Standard samples of Bis-GMA polymer were fabricated and used to implement compression test according to ASTM D695 standard test protocol. The nano-indentation experiments were conducted to verify the hyperelastic behavior of Bis-GMA polymer within the elastic limit. An FE model of the test was developed to assess the mechanical behavior and structural response of the Bis-GMA polymer using different hyperelastic constitutive models including Arruda–Boyce, Polynomial, Reduced polynomial, Ogden, Yeoh, and Van der Waals models. A hybrid experimental-computational approach was developed to link the experiment and simulation data to develop a validated FE model, as well as to analyze the internal behavior of the Bis-GMA polymer represented by different hyperelastic models. The stress–strain behavior, structural deformation, and the response of Bis-GMA polymer were used as the indicator to assess the hyperelastic behavior. Results showed that the maximum nominal strain value in which the material behaves hyperelasticly was equal to 0.3 mm/mm. Moreover, the second order polynomial hyperelastic model was obtained as the best fit to represent the mechanical behavior of Bis-GMA polymer. The validated FE model incorporating the second order polynomial hyperelastic model was used to obtain the true stress–strain curve of Bis-GMA polymer. The knowledge to obtain the constitutive model and mechanical behavior of polymer materials is essential for the design and mechanical analysis of polymer-based materials and structures. In this respect, the hybrid experimental-computational approach presented in this study is recommended for the mechanical characterization and to obtain the constitutive models and engineering/true stress–strain response of other polymeric materials. The approach is also suggested to be used for the determination of the mechanical behavior of polymers including Bis-GMA under different types of quasi-static load including tensile, shear, etc. 

## Figures and Tables

**Table 1:** The strain energy potential models used in the simulation of Bis-GMA polymer under monotonic compressive load.

Model Name	Equation	Detail
**Arruda-Boyce form [47]**	$\begin{array}{l} U=\mu \{\frac{1}{2}({\bar{I}}_{1}-3)+\frac{1}{20\lambda_{m}^{2}}({{\bar{I}}_{1}}^{2}-9)+\frac{11}{1050\lambda_{m}^{4}}({{\bar{I}}_{1}}^{3}-27)\\ +\frac{19}{7000\lambda_{m}^{6}}({{\bar{I}}_{1}}^{4}-81)+\frac{519}{673750\lambda_{m}^{8}}({{\bar{I}}_{1}}^{5}-243\}+\frac{1}{D}\left(\frac{J_{el}^{2}-1}{2}-\ln J_{el}\right) \end{array}$	-
**Polynomial form [48]**	$U=\sum_{i+j=1}^{N}C_{ij}{\left({\bar{I}}_{1}-3\right)}^{i}{\left({\bar{I}}_{2}-3\right)}^{j}+\sum_{i=1}^{N}\frac{1}{D_{i}}(J_{el}-1{)}^{2i}$	N = 1, 2
**Reduced polynomial form [49]**	$U=\sum_{i=1}^{N}C_{i0}{\left({\bar{I}}_{1}-3\right)}^{i}+\sum_{i=1}^{N}\frac{1}{D_{i}}(J_{el}-1{)}^{2i}$	N = 1, 2, …, 6
**Ogden form [50]**	$U=\sum_{i=1}^{N}\frac{2\mu_{i}}{\alpha_{i}^{2}}({\bar{\lambda }}_{1}^{\alpha_{i}}+{\bar{\lambda }}_{2}^{\alpha_{i}}+{\bar{\lambda }}_{3}^{\alpha_{i}}-3)+\sum_{i=1}^{N}\frac{1}{D_{i}}(J_{el}-1{)}^{2i}$	N = 1, 2, …, 6
**Yeoh form [51]**	$\begin{array}{l} U=C_{10}({\bar{I}}_{1}-3)+C_{20}{\left({\bar{I}}_{1}-3\right)}^{2}+C_{30}{\left({\bar{I}}_{1}-3\right)}^{3}\\ +\frac{1}{D_{1}}{\left(J_{el}-1\right)}^{2}+\frac{1}{D_{2}}{\left(J_{el}-1\right)}^{4}+\frac{1}{D_{3}}{\left(J_{el}-1\right)}^{6} \end{array}$	Reduced polynomial N = 3
**Van der Waals form [52]**	$U=\mu \left\{-(\lambda_{m}^{2}-3)\left[\ln(1-\eta )+\eta \right]-\frac{2}{3}a{\left(\frac{\bar{I}-3}{2}\right)}^{\frac{3}{2}}\right\}+\frac{1}{D}\left(\frac{J_{el}^{2}-1}{2}-\ln J_{el}\right)$	-

**Table 2:** Chemical characteristics of the materials used to prepare Bis-GMA polymer.

Commercial Name	Chemical Name	Molecular Formula	Molecular Weight (g/mol)	Manufacturer
**Bis-GMA**	2,2-bis[4-(2-hydroxy-3-methacryloxypropoxy)phenyl propane	C_29_H_36_O_8_	512.59	Sigma-Aldrich Inc., St. Louis, MO, USA
**Camphorquinone**	2,3-bornadenione	C_10_H_14_O_2_	166	Sigma-Aldrich Inc., St. Louis, MO, USA
**DMAEMA**	2-(dimethylamino) ethyl methacrylate	C_7_H_14_NO_2_	157	Sigma-Aldrich Inc., St. Louis, MO, USA
